# Hardware-Intrinsic Physical Unclonable Functions by Harnessing Nonlinear Conductance Variation in Oxide Semiconductor-Based Diode

**DOI:** 10.3390/nano13040675

**Published:** 2023-02-09

**Authors:** Namju Kim, Seung-Bae Jeon, Byung Chul Jang

**Affiliations:** 1 School of Electronic and Electrical Engineering, Kyungpook National University, 80 Daehakro, Bukgu, Daegu 41566, Republic of Korea; 2 Department of Electronic Engineering, Hanbat National University, 125 Dongseo-daero, Yuseong-gu, Daejeon 34158, Republic of Korea; 3 School of Electronics Engineering, Kyungpook National University, 80 Daehakro, Bukgu, Daegu 41566, Republic of Korea

**Keywords:** IoT, physical unclonable function, oxide semiconductor, Schottky diode

## Abstract

With the advancement of the Internet of Things (IoT), numerous electronic devices are connected to each other and exchange a vast amount of data via the Internet. As the number of connected devices increases, security concerns have become more significant. As one of the potential solutions for security issues, hardware intrinsic physical unclonable functions (PUFs) are emerging semiconductor devices that exploit inherent randomness generated during the manufacturing process. The unclonable security key generated from PUF can address the inherent limitations of conventional electronic systems which depend solely on software. Although numerous PUFs based on the emerging memory devices requiring switching operations have been proposed, achieving hardware intrinsic PUF with low power consumption remains a key challenge. Here, we demonstrate that the process-induced nonlinear conductance variations of oxide semiconductor-based Schottky diodes provide a suitable source of entropy for the implementation of PUF without switching operation. Using a mild oxygen plasma treatment, the surface electron accumulation layer that forms naturally in oxide semiconductor film can be partially eliminated, resulting in a large variation of nonlinearity as an exotic entropy source. The mild plasma-treated Schottky diodes showed near ideal 50% average uniformity and uniqueness, as well as an ideal entropy value without the need for additional hardware area and power costs. These findings will pave the way for the development of hardware intrinsic PUFs to realize energy-efficient cryptographic hardware.

## 1. Introduction

Advances in Internet of Things (IoT) technologies have driven a paradigm shift from processing-centric computing to memory-centric computing, resulting in a massive influx of digitally stored data. The IoT edge devices generate, store, and communicate massive amounts of digital information through interconnected networks and devices [1,2]. Considering that IoT devices are utilized in various applications such as healthcare [3], railway transportation systems [4], and convergence with artificial intelligence services [5], they process a vast volume of sensitive data which are susceptible to hacking and cyberattacks. In particular, with the proliferation of IoT edge devices, there has been a significant increase in the amount of data that devices must process and store through remote networks. This has made it imperative to develop new and advanced security protocols and cryptography systems. This is because the huge amount of data being transferred and stored through these networks makes them vulnerable to cyber threats. However, conventional cryptographic solutions that rely entirely on software have limitations in addressing the rising cybercriminal threat. For encryption and access authentication, conventional cryptographic methods depend on secret keys stored in nonvolatile memory. The attacker must have access to these security keys in order to apply Kerckhoff’s principle and Shannon’s maxim to decrypt any encryption system; hence, it is important that they are processed safely [6]. However, this security key storage is highly susceptible to physical and side channeling attacks by direct probing and power analysis [7,8]. Therefore, the cryptographic community has recently become interested in developing cryptographic systems integrated with hardware intrinsic PUF.

Physical unclonable functions (PUFs) are being spotlighted as the most promising hardware cryptographic primitives because they can generate a unique and random security keys on demand [9]. These security keys are generated by harnessing the inherent variations in the manufacturing process or the stochastic physical mechanism of electronic devices. The security keys are stored internally without the use of the external device and are not generated by other PUF devices, indicating that the unclonable digital security keys are analogous to a unique human fingerprint. These PUFs can be represented mathematically as a function that maps input challenges to output responses. A specific challenge and its corresponding response are called a challenge–response pair (CRP), which can be used as a security key and as a source for generating truly random numbers [10].

A precursor to the PUF, which utilized unclonable physical disorder, was the unique object [9]. During the Cold War, light-reflecting particles were sprayed on the exterior of nuclear weapons as a means of weapon arms control [11]. The random distribution of these particles created a unique and unpredictable interference pattern when the weapons were illuminated, making them difficult to clone. In this PUF, the angle of illumination is considered as the challenge and the 2D speckle pattern is its corresponding response. Current state-of-the-art PUFs mainly utilize complementary metal oxide semiconductor (CMOS) technologies that exploit the frequency of ring oscillators [12,13,14], the gate delay in multiplexers [15,16,17], and the stochastic initial state of static random access memory [18,19,20,21]. However, most CMOS-based PUFs suffer from not only high power consumption, poor entropy, and large-area overhead, but also vulnerability to machine learning attacks [6,9,10,22]. In addition, they require additional error correcting pre- and/or post-processing units to compenstate small device-to-device mismatches for reliable PUF opeation [10,23]. The challenges of CMOS-based PUFs turned the attention of reseachers to the emerging nanoelectdrode devices with the attractive features: memristive PUFs [6,24,25], carbon nanotube PUFs [26,27,28], and graphene PUFs [10]. Among these PUFs, the most promising PUF is memristive PUF with crossbar array architecture due to its simple structure, relatively low cost process, and small footprint. However, the memristive PUFs utilize the device-to-device variations generated by the threshold switching which results in an abrupt change of conductance when a large voltage or current is applied. Furthermore, memristive PUFs have limitations regarding the number of times they can be written or erased before they become inoperable, because they rely on the write and erase operations for key generation. Recently, a memristive PUF was developed that utilizes device nonlinearity as a source of entropy [24]. Considering that the reliable operation of memristor requires a sophisticated stoichiometry of resistive switching material, the development of a new device is necessary to exploit the nonlinearity of the device for realization of hardware intrinsic PUF.

In this study, we show that Schottky diodes based on an amorphous oxide semiconductor can utilize the device nonlinearity to implement PUF. We selected the amorphous In-Zn-Sn-O (a-IZTO) material instead of the commonly used amorphous In-Ga-Zn-O (a-IGZO) in the display industry because a-IZTO material has higher electron mobility (20–30 cm^2^/Vs) compared to a-IGZO [29]. To increase the variation in nonlinearity, we partially removed the naturally formed surface electron accumulation layer (SEAL) in a-IZTO using a mild oxygen plasma treatment. The oxygen plasma-treated a-IZTO-Schottky diodes with Pd/A-IZTO/Pd structure showed random nonlinearity in their I-V characteristic with high entropy. The a-IZTO-Schottky diode-based PUF, called Schottky-PUF, demonstrated the ideal PUF characteristics, such as near ideal 50% average uniformity, uniqueness, and an ideal entropy, without the need for device switching, which leads to high power consumption. This makes the Schottky-PUF an energy-efficient PUF device compared to other PUF devices that require device switching.

## 2. Materials and Methods

The Schottky-PUF devices were fabricated by forming a crossbar array with the following structure: Pd/a-IZTO/Pd. To fabricate the Schottky-PUF devices, 60 nm-thick Pd electrodes were formed on a SiO_2_/Si substrate using a metal shadow mask and a thermal evaporation technique. After that, a 30 nm-thick a-IZTO film was deposited on the bottom Pd electrodes through radio-frequency (RF) sputtering under high vacuum conditions (0.088 Pa). The oxygen plasma was then treated using an inductively coupled plasma device to partially eliminate the SEAL in the a-IZTO film. Note that this oxygen plasma treatment process should be applied before formation of top electrodes. The final step involved depositing 60 nm-thick Pd top electrodes using thermal evaporation, which were perpendicular to the Pd bottom electrodes and had identical 60 μm widths, to form the crossbar array. Both Pd electrodes had identical 60 μm widths. The Schottky-PUF devices were electrically measured by applying a voltage to the Pd top electrode while the Pd bottom electrode was grounded. The electrical characterization of the Schottky-PUF was performed with a probe station using a Keithley 4200 semiconductor parameter analyzer in an air environment. It is noted that the a-IZTO-Schottky diode was characterized at room temperature in air environment without passivation layer, indicating reliable stability against moisture in air. The transmission electron microscopy (TEM) images were captured with JEOL ARM 300F equipment and the TEM sample was created using a focused ion beam (FIB) for cross-sectional analysis (FIB, FEI Helios Nano Lab 450 HP). The film characterization of a-ITZO film was performed using X-ray photoelectron spectroscopy (XPS). The XPS spectra were obtained via the Sigma probe multipurpose spectrometer (Thermo Fisher Scientific, Wlatham, MA, USA) with monochromatized Al Kα source in order to anlayze the chemical composition of a-IZTO with and without oxygen plasma treatment.

## 3. Results and Discussion

**Figure 1:**
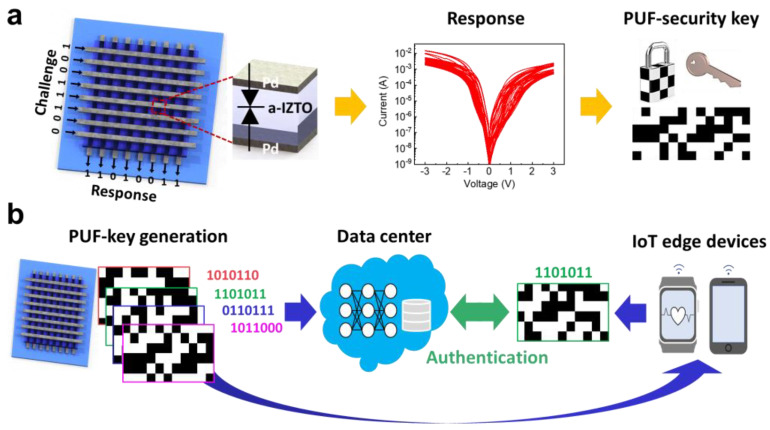
(**a**) Schottky-PUF based on a-IZTO-Schottky diode. The Schottky-PUF utilizes device nonlinearity to generate the security keys. (**b**) Concept of authentication process for accessing IoT edge device using Schottky-PUF.

Figure 1a shows how the nonlinearity of Schottky diodes can be used to generate CRPs as security keys in the Schottky-PUF, which is constructed with a crossbar array architecture. The Schottky-PUFs are fabricated using a simple structure that consists of Pd/a-IZTO/Pd organized in the crossbar array configuration. The back-to-back Schottky diodes based on the a-IZTO film exhibit the nonlinear current-voltage (I-V) characteristics in both the positive and negative voltage regions, indicating the formation of the Schottky barrier at both the top and bottom interfaces. To leverage the variation in nonlinearity as the source of entropy, we achieved a change in the Schottky barrier by partially removing the naturally formed SEAL in a-IZTO using the oxygen plasma treatment process. Note that the SEAL results in a reduction in the Schottky barrier width, i.e., effective Schottky barrier height, as will be discussed in more detail. As shown in Figure 1a, the highly variable nonlinearity of Schottky diodes indicates that the partial elimination of the SEAL via the mild oxygen plasma treatment process occurred differently for each device in the same crossbar array. To generate a security key with the Schottky-PUF, the input voltages are applied to the top electrodes of Schottky-PUF, which generates unique output response. The combination of the input voltages is referred to as a challenge, and the resulting response is labeled as a response. The CRPs generated from the Schottky-PUF can be used for the authentication process. The IoT edge devices requires the authentication process for end users using the personal information stored in cloud servers. However, the authentication process can still be exposed to malicious attacks during the transmission of data required for authentication between the IoT edge devices and cloud servers. These malicious cyber attacks such as hacking eavesdrop on and steal personal information to withdraw money from personal accounts. This security threat can be resolved using a highly secure cryptography system integrated with PUF devices. Figure 1b shows a representative example of our proposed protocol for authentication process using PUF devices. The CRPs generated by the Schottky-PUFs should be sent to the data center and the Schottky-PUFs are integrated with the IoT edge devices. Before using the edge device, the end user must enroll the CRPs generated by the edge device into the data center. Then, whenever an end user tries to access the edge device, the authentication process is performed by comparing one of the CRPs generated by the edge device with the CRPs that have been enrolled in the data center. The previously used CRP from the edge device should not be reused as it poses a security risk. This approach allows the Schottky-PUF to guarantee the confidentiality of the end user’s personal information when using IoT edge devices.

**Figure 2:**
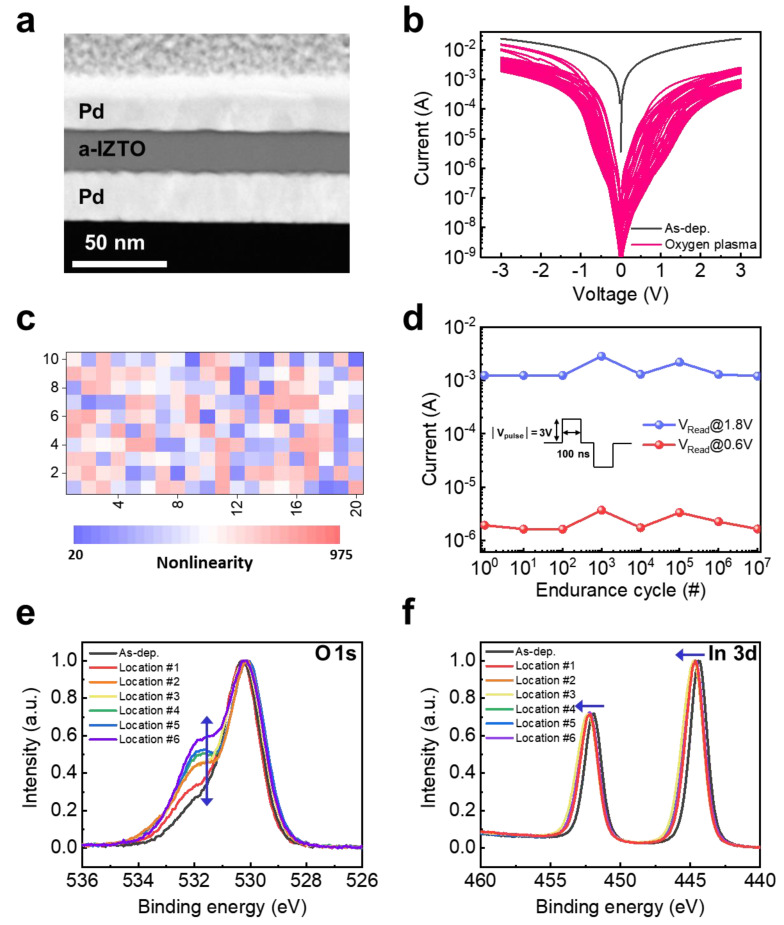
(**a**) The cross-sectional TEM image of a-IZTO-Schottky diode. (**b**) I-V characteristics of a-IZTO-Schottky diode. (**c**) Two-dimensional color map for device-to-device distribution of the device nonlinearity calculated from the current ratio measured at 1.8 V and 0.6 V. (**d**) Cycling endurance test results. XPS analysis of (**e**) O 1s core level and (**f**) In 3d core level spectra of a-IZTO films without and with oxygen plasma treatment.

Figure 2a shows a cross-sectional transmission electron microscopy (TEM) image of the fabricated a-IZTO-Schottky diode. It was confirmed that a-IZTO film with a thickness of 30 nm was uniformly formed on the Pd bottom electrode. We adopted the noble Pd electrode because its high work function (5.22 eV) [30] can form a high Schottky barrier height, considering the electron affinity of a-IZTO (4.5 eV) [31]. The noble Pd electrode also prevents the formation of an oxide layer with high bandgap at the interface with the a-IZTO film because the noble metal has a very high Gibbs free energy for oxide formation. On the other hand, the a-IZTO-Schottky diode based on as-deposited a-IZTO film showed nearly linear I-V characteristics (Figure 2b). Given that the SEAL is naturally formed by the reduction of the surface in a vacuum chamber [32,33,34,35], the nearly ohmic behavior indicates the presence of SEAL at the top surface of the a-IZTO film. A large number of studies have attempted to either eliminate the SEAL through the use of chemical surface treatments [36,37] and oxygen plasma treatment [34,35,38,39] or to prevent the formation of the SEAL through the deposition of a conductive polymer [40]. Among them, we adopted the oxygen plasma treatment process in order to increase the stochasticity of the Schottky barrier height, as its mild process condition cannot completely strip the SEAL of all Schottky diodes in the same crossbar array, resulting in random variation of the effective Schottky barrier heights between devices. In our previous work, it was confirmed that the generated oxygen plasma at RF 100 W power for more than 10 min completely eliminated the SEAL [31]. Thus, we used the generated oxygen plasma at 50 W power for 5 min to remove part of the SEAL. As shown in Figure 2b, the I-V characteristics of the oxygen plasma-treated a-IZTO-based Schottky diodes in the positive voltage region exhibit a higher degree of nonlinear variation compared to those in the negative voltage region. On the other hand, the as-deposited a-IZTO-based Schottky diode showed the highly linear I-V characteristic. Based on these results, it can be concluded that the Schottky barrier generated at the top surface is nonuniform because the SEAL was only partially removed. Figure 2c exhibits the 2D color map of the device-to-device distribution for nonlinearity of I-V characteristic. This 2D map distribution was extracted from eight arrays of 5 × 5 devices to illustrate the nonlinearity of I-V relationship. The device nonlinearity of I-V characteristics was calculated by the ratio of the current values measured at 1.8 V and 0.6 V. Figure 2d presents the results of the endurance test on the oxygen plasma-treated a-IZTO-Schottky diode. Despite 10^7^ cycles of pulse voltages with an amplitude of ±3 V and a width of 100 ns, the a-IZTO-Schottky diode exhibited no significant degradation of the window between the currents measured at 1.8 V and 0.6 V. The remarkable electrical stability of the a-IZTO-Schottky diode has great potential for the development of highly reliable PUF devices, because one of the figures of merit for PUF devices is reliability against electrical stress.

An X-ray photoelectron spectroscopy (XPS) study was performed to analyze the effect of the mild oxygen plasma treatment on the composition of the a-IZTO in Schottky-PUF. Figure 2e shows representative XPS spectra of the O 1s core level for a-IZTO film treated with and without the mild oxygen plasma treatement. The same mild oxygen plasma treatement was applied to all spots on the a-IZTO film, and a distinct change in the O 1s spectrum was observed. Each O 1s spectra has two peaks, one at 530 eV and the other at 531.9 eV. The O 1s peak at 530 eV belongs to M-O bonds (M-In, Zn, and Sn), whereas the other peak at 531.9 eV is the O-H, O-C, and O-O bonding adsorbed on the surface of a-IZTO [41,42,43]. The oxygen plasma treatment has no effect on the contaminated hydrogen and carbon species on the a-IZTO surface, because these species rapidly react with oxygen radicals and are eliminated during oxygen plasma treatment [44]. However, the variation in intensity for the O 1s peak with 531.9 eV can be attributed to variation in the O-O bonding of O^−^ ions absorbed on the a-IZTO surface, which indicates the formation of a nonuniform Schottky barrier across the a-IZTO film. In addition to O 1s spectra, the other binding energy peak of In 3d shifted toward higher binding energy when compared to that of the as-deposited a-IZTO film (Figure 2f). Other peaks, such as Sn 3d and Zn 2p, are also shifted toward the high binding energy. It is noted that the shift towards higher binding energy indicates that the surface of the a-IZTO film has been oxidized. These findings demonstrate that the moderate oxygen plasma treatment leads to the formation of the nonuniform Schottky barrier between Pd and a-IZTO, enabling the a-IZTO-Schottky diodes to be suitable for PUF applications by partially eliminating the SEAL.

**Figure 3:**
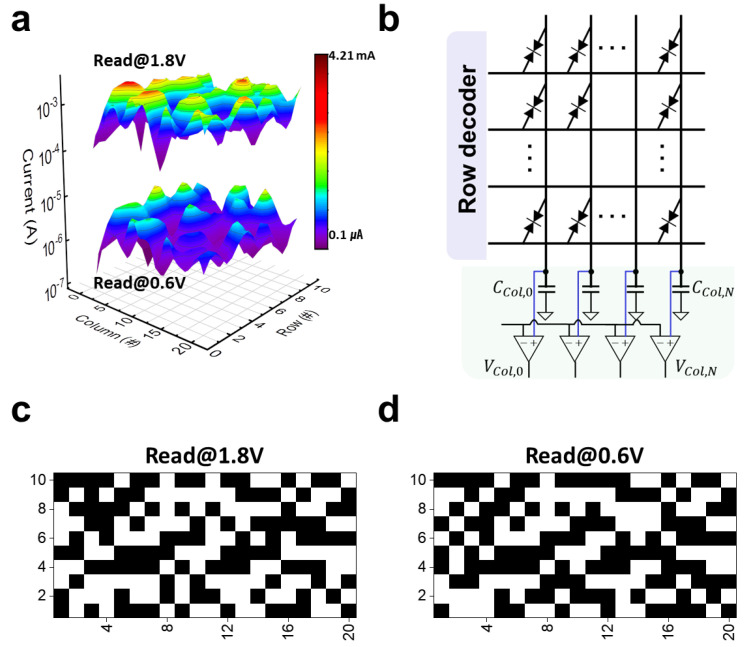
(**a**) Three-dimensional color map for device-to-device distribution of the currents measured at 1.8 V and 0.6 V. (**b**) Voltage sensing-based method to convert the analogue data to digital data. The converted digital data of the currents measures at (**c**) 1.8 V and (**d**) 0.6 V.

The 3D color map of device-to-device distribution for the currents measured at 1.8 V and 0.6 V is presented in Figure 3a. These currents were extracted from a total of 200 devices in eight crossbar arrays, which indicates a nonuniform distribution. To implement PUF function, we first transformed the analogue data into a digital data pattern using the reference voltage *V*_Ref_ as a basis. This digital pattern indicates a random distribution of 1′s and 0′s. As shown in Figure 3b, the Schottky-PUF-based digital security keys were generated by using a voltage sensing mode that was based on charge integration, as it consumes less power and occupies a smaller area of peripheral circuit than the current mode sensing [45]. In addition, the voltage sensing mode allows for sensing of very low current [45]. This is the main reason why the the commercialized NAND flash memory utilizes voltage sensing mode rather than current sensing mode [46]. Figure 3c,d show the digital bit maps that were converted from the currents measured at 1.8 V and 0.6 V, indicating the random idstribution of 1′s and 0′s. The digital states ‘1’ and ‘0’ are represented by white and black squares, respectively, and are determined based on whether the voltage accumulated by the current is greater or less than *V*_Ref_ by the median current of the devices. The median split technique is generally used to digitize the random analogue values in emerging device-based PUF [47,48]. The *V*_Ref_ was selected from one of the eight arrays, each consisting of 5 × 5 Schottky-diode devices, but it should be noted that it is the median value of only one array and not the median value of the remaining seven arrays. It is worth mentioning that the Schottky-PUF does not require switching operations that have high power consumption. Therefore, the Schottky-PUF has an advantage of low power consumption, which is particularly important as the emerging memory device-based PUFs consume a large amount of power for their switching operation.

**Figure 4:**
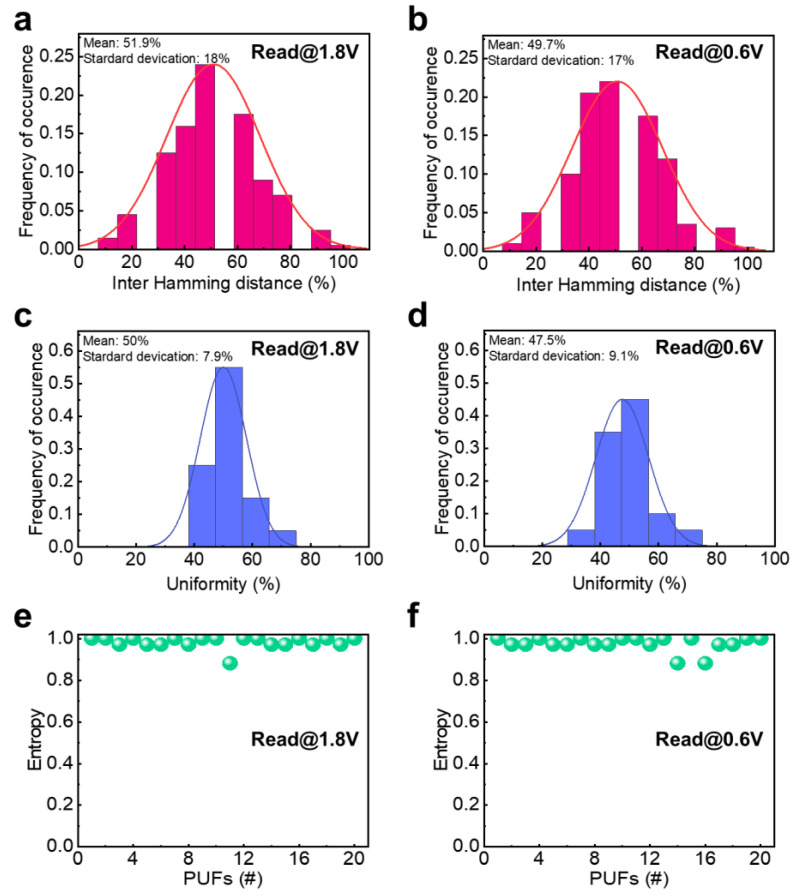
The results of the evaluated PUF key figures of merit of Schottky-PUF. Uniqueness measured at (**a**) 1.8 V and (**b**) 0.6 V read voltages. Uniformity measured at (**c**) 1.8 V and (**d**) 0.6 V read voltages. Entropy measured at (**e**) 1.8 V and (**f**) 0.6 V read voltages.

To evaluate the feasibilty and performance of the Schottky-PUF, we investigated two important parameters: the first was uniqueness, which was determined by the Hamming weight, and the second was uniqueness, determined by the inter-Hamming distance (HD) [49]. In order to generate one 20-bit-long CRP, we first created 10 groups, each of which contained 20 devices, from the eight arrays. The uniformity is calculated as the number of ‘1’ and ‘0’ in the response’s bit stream, i.e., the percentage of Hamming weight [50]. To achieve the ideal random responses, the mean distribution of uniqueness must be guaranteed to be 50%. For an n-bit response, the uniformity was calculated as a percentage of the Hamming weight as the following equation [49,50]:

$$Uniformity=\frac{1}{n}\overset{n}{\underset{i=1}{\sum }}b_{i}\times 100\;\%$$

where $b_{i}$ indicates the number of a bit ‘1’. As shown in Figure 4a,b, the Schottky-PUF showed 51.9% and 49.7% at 1.8 V and 0.6 V of read voltages, respectively. The uniformity values close to the ideal value indicate that the generated security key is unpredictable and unclonable, with minimal bias, implying the excellent uniqueness of the Schottky-PUF.

The uniqueness indicates how independent the responses generated by different PUFs with the identical challenge are from each other. This was determined by counting and comparing the number of distinct bits among the PUFs, i.e., the inter-HD. Thus, the inter-HD beween any two PUFs was computed as follows [49,50]:

$$Uniquness=\frac{2}{k\left(k-1\right)}\overset{k-1}{\underset{i-1}{\sum }}\overset{k}{\underset{j=i+1}{\sum }}\frac{HD\left(R_{i},\;R_{j}\right)}{n}\times 100\;\%$$

where $R_{i}$ and $R_{j}$ are the n-bit responses of the security key ‘*i*’ and ‘*j*’ for a given challenge, and *k* is the number of the security key and *n* is the length of the security key. The inter-HD of Schottky-PUF was calculated using statistical data obtained from 45 (_10_C_2_) pairs of CRPs, which were randomly selected two from 10 groups. Figure 4c,d illustrates that the inter-HD of Schottky-PUF was, on average, 50% ± 7.9% and 47.5% ± 9.1% at 1.8 V and 0.6 V read voltages, respectively. When the HD between two CPRs is too long or short, one CRP can be easily decrypted using the information of the other PUF, thus requiring an ideal value of 50% for the high security. The uniqueness of Schottky-PUF is clearly guaranteed, as their uniqueness values were close to the ideal value. In addition, the entropy, which is well known as a measure of the randomness of a system, was calculated as follows [10]:

$$E=-\left[p\log_{2}p+\left(1-p\right)log_{2}\left(1-p\right)\right]$$

where *p* is the uniformity of the PUF. The Schottky-PUF showed entropy values extremly close to the ideal value of one for both 1.8 V and 0.6 V of read voltages, respectively (Figure 4e,f). This indicates that they have unpredictable randomness. This near ideal entropy value reveals that the Schottky-PUF is capable of generating unique and random responses, making it highly suitable for securing sensitive data in various applications. Therefore, the achieved uniformity, uniqueness, and entropy, which are close to their ideal values, reveal the significant potential of the Schottky-PUF for realizing a highly secured electronic system.

## 4. Conclusions

We have reported that the a-IZTO-Schottky diodes can implement hardware intrinsic PUF. The mild oxygen plasma treatment results in nonuniform Schottky barrier on the top surface of the a-IZTO-Schottky diodes by partially removing the SEAL. The mild oxygen plasma-treated a-IZTO-Schottky diodes showed a wide range of nonlinear I-V characteristics, indicating the presence of a source with a high entropy content. The large variation in nonlinearity is attributed to the change in the amount of oxygen ions adsorbed on the a-IZTO surface, which was demonstrated via XPS analysis. The oxygen plasma-treated a-IZTO-Schottky diodes showed stable operation against a harsh cycling endurance test, demonstrating reliable Schottky-PUF against electric stress. In addition, the Schottky-PUF can generate the unclonable security keys with near perfect uniformity, uniqueness, and entropy characteristics. However, there remain issues to be solved for development of high-density PUF devices. One of the main hurdles is the effect of severe fabrication variation when scaling down to nm scale. This effect causes severe problems in the read margin due to the ultralow current-induced noise, as the current of the Schottky diode is proportional to the active device area due to its conduction mechanism based on thermionic emission. To address the limitation, a vertical three-dimensional (3D) structure such as the commercialized 3D NAND Flash memory can be the most promising solution; we plan to study this topic as our further work. Therefore, the Schottky-PUF with high-density 3D structure could pave the way for the development of built-in hardware cryptographic systems for data-intensive applications such as IoT edge devices.

## Data Availability

Not applicable.
